# Clinical and Radiographic Outcomes of Locking-Plate Fixation Augmented with a Porous Hydroxyapatite Bone Substitute for Proximal Humerus Fractures: A Retrospective Cohort Study with 12-Month Follow-Up

**DOI:** 10.3390/jfb17060279

**Published:** 2026-06-05

**Authors:** Achille Saracco, Leo Massari, Marco Amadio, Riccardo Menin, Gaetano Caruso

**Affiliations:** 1 Department of Translational Medicine and for Romagna, University of Ferrara, c/o “S. Anna”, Via Aldo Moro 8, 44124 Ferrara, Italy; 2 Department of Neurosciences and Rehabilitation, University of Ferrara, c/o “S. Anna”, Via Aldo Moro 8, 44124 Ferrara, Italy; marco.amadio@ospfe.it (M.A.); riccardo03.menin@edu.unife.it (R.M.); gaetano.caruso@unife.it (G.C.)

**Keywords:** augment, biomaterials, bone substitutes, trauma, shoulder, humerus

## Abstract

Background: Evidence on the role of synthetic biomimetic bone substitutes in the surgical management of proximal humerus fractures remains limited. This study aimed to evaluate the clinical, radiographic, and safety outcomes of a porous hydroxyapatite bone substitute used as an adjunct to locking-plate fixation in proximal humerus fractures with metaphyseal bone loss. Methods: We performed a retrospective comparative cohort study including 45 patients treated with locking-plate fixation and porous hydroxyapatite scaffold augmentation and 40 comparable control patients treated with locking-plate fixation without scaffold augmentation. Patients were evaluated clinically and radiographically at 1, 3, 6, and 12 months after surgery. Functional outcome was assessed with the Constant–Murley Score (CMS), and pain was assessed using the Visual Analogue Scale (VAS). Longitudinal changes over time were analyzed using mixed-effects models for repeated measures. Results: CMS improved progressively over follow-up, whereas VAS pain scores decreased significantly over time. No cases of device migration or radiographic resorption were observed during follow-up. Adverse events were recorded, but no complication was considered directly attributable to the implanted biomaterial. Functional recovery and pain reduction followed a similar trajectory in both groups, with no significant group-by-time interaction. Conclusions: In this retrospective series, graft augmentation with a porous hydroxyapatite scaffold during locking-plate fixation of proximal humerus fractures with bone void was associated with progressive functional improvement and pain reduction, without evident device-related safety concerns. Owing to the retrospective, non-randomized design, limited sample size, potential selection bias, and incomplete follow-up in part of the cohort, these findings should be interpreted as supportive of feasibility and short- to mid-term safety rather than as definitive evidence of biomaterial efficacy. Level of Evidence: Level III, retrospective cohort study.

## 1. Introduction

The use of biomimetic bone substitutes has markedly increased over the last decade, becoming a routine component of surgical practice. Their introduction has contributed to a steady decline in the use of autologous bone grafts, which are unavoidably associated with donor-site morbidity [1]. Although autologous grafts remain the biomechanical gold standard, biomimetic substitutes have produced increasingly promising outcomes. As a result, a wide range of devices composed of different biomaterials and available in various configurations are now commercially accessible [2]. Among the most employed materials are hydroxyapatite, tricalcium phosphate, calcium phosphate, and calcium sulfate, each offering distinct osteoinductive, osteoconductive, osteogenic, and load-bearing properties. These characteristics have favored their widespread adoption in several anatomical regions [3].

Despite their growing popularity and reassuring safety profiles [4], the literature still provides limited evidence on their effectiveness across different clinical applications. Most studies originate from the orthopedic domain [5], whereas data specific to traumatology remain comparatively scarce.

This investigation explores the use of a device originally engineered for a specific anatomical region—the proximal humerus—in the surgical management of complex fractures. Proximal humeral fractures constitute the third most common fracture of the upper limb [6], with a predominance in elderly women. Population aging is contributing to a rising incidence of complex fracture patterns and a consequent increase in surgical indications [7].

In the present study, we examined a cohort of patients who underwent osteosynthesis with open reduction and internal fixation (ORIF) for proximal humeral fractures [8]. In this retrospective comparative cohort study, we evaluated patients treated with locking-plate fixation with or without adjunctive hydroxyapatite scaffold augmentation [9]. Each fracture demonstrated a minimum bone void of 1 cm^3^; in selected cases, the use of a filler material was considered useful to enhance the biomechanical environment and support bone healing. Hydroxyapatite (HA) is a mineral component naturally present within bone tissue. The porous structure of the device enables effective chemical interaction with the surrounding tissues, thereby supporting graft functionality at the implantation site. This material has demonstrated favorable performance with respect to the biophysical characteristics described above, while also offering a cost advantage.

Although the load-bearing capacity of hydroxyapatite is inferior to that of other biomaterials, it provides superior osteoconductive and osteointegrative properties [10,11]. In recent years, alongside advances in biomaterial composition, device design has also been optimized to meet diverse surgical requirements through the development of markedly different configurations.

**Figure 1:**
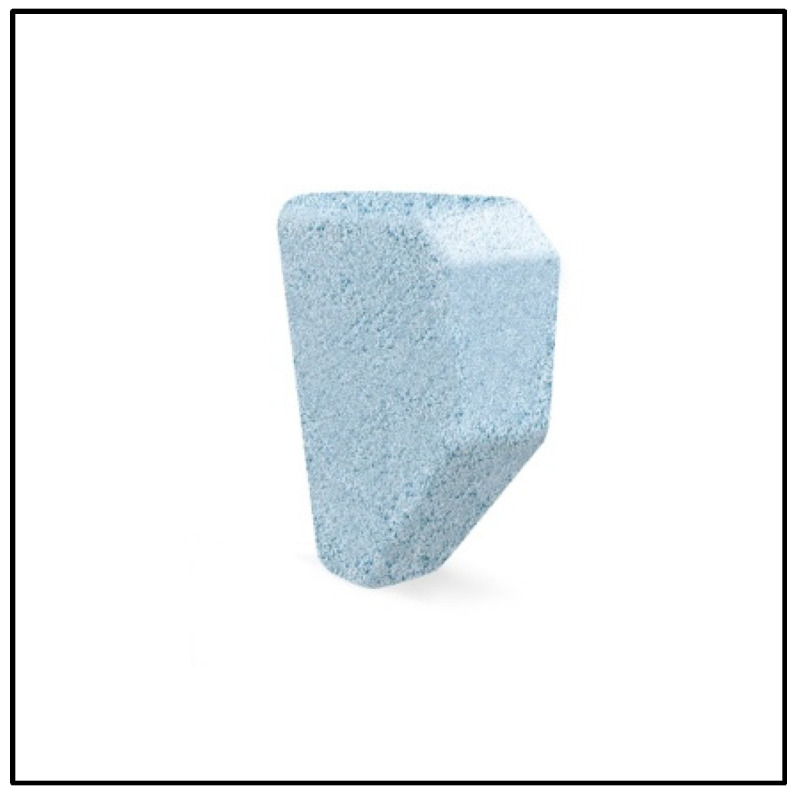
The biomaterial device in the SH conformation, prepared for the proximal humerus. We thank Fin-Ceramica SpA, Faenza RA, Italy, for their permission to use the image.

In our cohort, we used the product ENGIpore (Fin-Ceramica S.p.A., Faenza, RA, Italy), a preformed bone substitute in its SH configuration (Figure 1), which is specifically designed for the proximal humerus, namely the humeral head. According to the manufacturer, “This product is an implantable, resorbable, non-active medical device acting as a bone substitute. It consists of biomimetic porous hydroxyapatite. These medical devices are characterized by extremely high porosity, reaching up to 90% of the total volume. Their interconnected pore system is composed primarily of macro- and micro-pores. The macro-pores typically range from 200 to 500 μm, while the interconnected micro-pores range from 80 to 200 μm. In addition, an intergranular microporosity (<10 μm) facilitates the absorption of physiological fluids. This architecture optimizes cellular colonization by bone-regenerating cell populations, thereby ensuring effective osteointegration and osteoconduction” [12]. The device consists exclusively of the biomaterial itself; therefore, it is not supplemented with drugs or other substances. Furthermore, it does not undergo any specific surface treatments.

An additional relevant characteristic is the device’s prolonged remodeling time: structural modifications generally become evident approximately 24 months following implantation.

The advantages of this device are:

- Ready-to-use product;
- Safety profile and lower infectious risk than autologous [13];
- No need to plan operating field for autologous graft;
- Availability of different device sizes and simple adaptation of shape and size.

Conducting efficacy studies on such devices is challenging because the endpoints to be assessed may vary according to the anatomical region considered in the literature. In traumatology, it is essential to evaluate the behavior of the graft in relation to the biological healing process. For this reason, we carried out meticulous radiographic and clinical assessments. The investigation focused primarily on potential device extrusion, migration, or delays in fracture consolidation.

The aim was to evaluate, through clinical and radiographic follow-up, postoperative recovery and the biomechanical stability of fractures characterized by a substantial bone defect treated with biomimetic bone grafting. Accordingly, a cohort of patients was selected with comparable characteristics, similar fracture patterns, and identical surgical management, to optimize the assessment of device interactions [14].

## 2. Materials and Methods

### 2.1. Study Design and Ethical Approval

We conducted a retrospective comparative cohort study on a population of 45 patients treated surgically at “University Hospital of Ferrara; Orthopaedics and Traumatology Unit” for proximal humeral fractures.

The study period extended from January 2022 to January 2025 to allow for adequate follow-up. The aim of this retrospective comparative cohort study was to compare the clinical, radiographic, and safety outcomes of locking-plate fixation with versus without porous hydroxyapatite scaffold augmentation in proximal humerus fractures with metaphyseal bone loss. To assess the safety and effectiveness of the biomaterial, a comparison group of patients with comparable clinical characteristics who underwent the same surgical treatment without biomaterial augmentation was included.

The study involved a comparison between two groups with similar proximal humerus fractures, bone loss, and homogeneous baseline characteristics. The control group consisted of patients with comparable fracture patterns and metaphyseal bone loss who underwent the same fixation strategy without scaffold augmentation, according to intraoperative surgeon assessment. Not all patients underwent a preoperative CT scan; therefore, in some cases, the measurement of the bone gap may have been less precise. In all cases, however, the presence of bone loss was identified and documented in the operative report by an experienced surgeon.

This study was reviewed and granted ethical approval by AVEC, Italy (IT), in February 2026 (707/2025/Oss/AOUFe evaluated in the session of 18 February 2026). The research methodology, including data collection, participant recruitment, and all associated procedures, was designed and conducted in strict accordance with ethical standards.

### 2.2. Patient Selection: Inclusion and Exclusion Criteria

**Figure 2:**
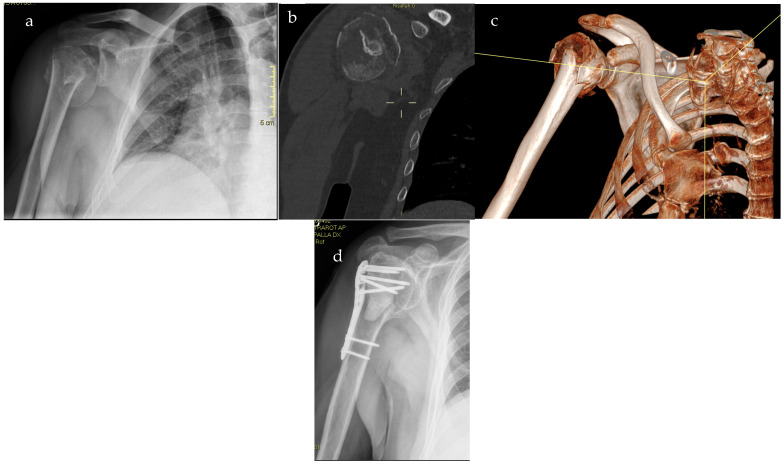
(**a**) Radiographic images; CT scan (**b**) and 3D CT (**c**) reconstruction of fracture with varus breakdown and bone gap. (**d**) Post-operative evaluation; the presence of the device replaces the loss of substance.

**Figure 3:**
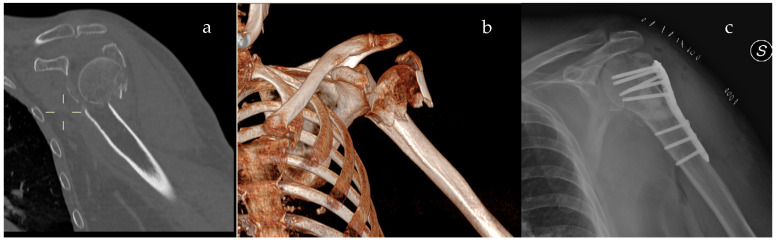
(**a**) Radiographic images and (**b**) 3D CT reconstruction of fracture with valgus breakdown and bone gap. On the right, (**c**) post-operative evaluation.

All patients included in the study were admitted to the Trauma Emergency Department, where preoperative clinical assessment and initial radiographic evaluation were performed. In some cases, an investigation using CT scan was requested to evaluate the correct surgical indication. They were subsequently admitted to the inpatient ward to await the surgical procedure (Figure 2 and Figure 3). The fractures were initially classified according to the AO/OTA (AO Foundation/Orthopaedic Trauma Association) classification and the Neer classification. All included fractures presented a significant bone gap, with a minimum bone void of 1 cm^3^.

Eligibility criteria were as follows:

- Age ≥ 18 years;
- Presence of fractures of the proximal portion of the humerus from trauma occurring no more than 15 days previously;
- Treatment with open reduction and internal fixation (ORIF);
- Intervention performed by the same trauma team;
- No open fracture in the treated portion;
- Exclusion of nervous or vascular deficits;
- Minimum bone void of 1 cm^3^.

Exclusion criteria were as follows:

- No bone void or bone void < 1 cm^3^;
- Pediatric fractures;
- Fracture patterns not meeting the predefined AO/OTA or Neer classification criteria;
- Fractures with exposed bone;
- Neurovascular deficit.

### 2.3. Biomaterial and Implanted Devices

To address bone void, a pre-formed Engipore^®^ SH bone substitute (Fin-Ceramica S.p.A., Faenza, RA, Italy) was implanted. In our clinical practice, the device used for this fracture type was the SH configuration, specifically designed for the proximal humerus region. This device can be used either in its original configuration or shaped as needed without compromising its properties. The choice of how to use it was left entirely to the surgeon during the surgical procedure. Whenever it was necessary to fill the bone gap after fracture reduction, the device was placed and adapted to the specific defect. The device is commercially available in different sizes. In our clinical practice, we used the small and large sizes.

### 2.4. Surgical Procedure

The surgical procedures were performed by six orthopedic surgeons specialized in upper-limb traumatology, using either the deltopectoral or the transdeltoid surgical approach. In the latter case, a minimally invasive approach and the application of the MIPO (Minimally Invasive Plate Osteosynthesis) technique were employed to achieve reduction and fixation. The possible use of the biomaterial device was determined after fracture reduction and before plate fixation. All surgeries were performed under general anesthesia with the patient positioned in the Beach-Chair position. All patients were treated with either the PHILOS or PHILOS Long 3.5 mm LCP Proximal Humerus Plate (DePuy Synthes^®^, J&J MedTech, New Brunswick, NJ, USA). At the end of the procedure, all patients were immobilized with an arm sling until suture removal.

### 2.5. Postoperative Management and Follow-Up

After suture removal, patients began the rehabilitation program. During the first week after suture removal, active pendular exercises and passive mobilization were allowed. From the following weeks onward, range of motion and functional effort were progressively increased according to the physiotherapist’s judgment. Four weeks after the procedure, the sling was completely discontinued.

Patients were followed clinically and radiographically at 1, 3, 6, and 12 months after surgery. Clinical evaluations were performed by the same medical team that conducted the surgical procedures.

Radiographic assessments were performed using DICOM-based Carestream Vue PACS software, version 11.4 (Carestream Health, Inc., Rochester, NY, USA), allowing multiplanar evaluation of the treated proximal humerus. The radiographic analysis focused on secondary articular surface changes, disruption of the medial humeral hinge, secondary displacement of the tuberosities, and collapse of the humeral head/epiphyseal segment. The occurrence of any of these findings was classified as secondary loss of reduction. Conversely, the presence of epiphyseal or metaphyseal bone resorption was classified as necrosis or early radiographic signs of humeral head osteonecrosis. During clinical follow-up, several parameters were assessed, including joint range of motion recovery, limb muscle strength, resumption of daily activities, and symptoms reported both at rest and during movement. Any clinical complications were documented. This assessment was performed by both the radiologist and the orthopedic surgeon. Radiographic assessments were performed independently by two evaluators. In cases of disagreement, the images were reviewed by a third senior evaluator, whose judgment was considered final. Radiographic imaging was simultaneously reviewed to evaluate bone healing and device performance. Attention was given to potential bone resorption, device extrusion, humeral head collapse, and secondary fracture displacement (Figure 2 and Figure 3).

Clinical evaluation was performed on an outpatient basis by an orthopedic surgeon with expertise in upper-limb trauma. The Constant–Murley Score (CMS) was used to quantify and objectively evaluate clinical outcomes. This widely used shoulder assessment tool comprises four components—pain, activities of daily living, active range of motion, and strength—yielding a maximum score of 100 points. Higher scores indicate better shoulder function. Additionally, the Visual Analogue Scale (VAS) was used to assess pain severity through a patient-reported visual scoring method.

### 2.6. Statistical Analysis

All data were entered into a database created using Microsoft Excel (Redmond, Washington, DC, USA) and analyzed with STATA Version 12 (StataCorp, College Station, TX, USA).

Statistical analysis was performed to compare two clinically homogeneous groups of patients with proximal humerus fractures treated with the same reduction strategy, the same locking-plate fixation system, and the same surgical team. The two groups were selected to ensure comparable baseline demographic, fracture-related, and surgical characteristics, with the use of the biomaterial device representing the main treatment-related difference between cohorts. Continuous variables were assessed for normality using the Shapiro–Wilk test. Normally distributed variables are reported as mean ± standard deviation (SD) and were compared between groups using Student’s *t*-test for independent samples. Non-normally distributed variables are reported as median and interquartile range (IQR) and were compared using the Mann–Whitney U test. Categorical variables are reported as counts and percentages and were compared using the chi-square test or Fisher’s exact test, as appropriate. For longitudinal clinical outcomes, the Constant–Murley Score (CMS), which showed an approximately normal distribution, was analyzed using a linear mixed-effects model with group, time, and group-by-time interaction as fixed effects and patient as a random effect. VAS pain scores showed a non-normal distribution and were summarized as median [IQR]; between-group comparisons at each follow-up time point were performed using the Mann–Whitney U test. Longitudinal changes in VAS were assessed using a mixed-effects model, supported by non-parametric sensitivity analysis restricted to patients with complete follow-up data using the Friedman test. A two-sided *p* value < 0.05 was considered statistically significant.

## 3. Results

The final study population included 45 patients (27 female; 18 male).

The control group, identified based on comparable characteristics and a similar treatment timeframe, consisted of 40 patients (23 women and 17 men).

The mean age was 67.0 ± 8.7 years in the study group and 65.0 ± 7.5 years in the control group.

The anesthetic risk was quantified by the ASA (American Society of Anesthesiologists) score, with 14 patients ranked ASA 2, 24 patients ASA 3 and 7 patient ASA 4. In the control group 15 patients were ranked ASA 2, 19 patients ASA 3 and 6 patients ASA 4.

In 23 patients, the right upper limb was involved, while in the remaining 22 patients, the left limb was involved. In 25 cases, the dominant limb was involved.

Before the surgical procedure, a first diagnostic radiography was performed in several projections of the proximal humerus. A preoperative shoulder CT scan was also available in 30 patients in the study group. In the control group, a CT scan was available in 23 cases.

The fracture type was classified by AO/OTA and Neer classification, obtaining:

- 25 AO/OTA 11B fractures;
- 20 AO/OTA 11 C fractures;
- 24 Neer 3-part fractures;
- 21 Neer 4-part fractures.

Surgical procedures were performed by six orthopedic surgeons with expertise in trauma surgery. In 27 cases, the deltoid–pectoral approach was chosen, while in 18 cases, the fracture was managed by a trans-deltoid approach and MIPO (Minimally Invasive Plate Osteosynthesis) technique. The two groups, when compared, showed homogeneous characteristics, with differences that were not statistically significant (*p*-value > 0.05).

Table 1 summarizes the characteristics of population.

Fixation was performed using PHILOS or PHILOS Long 3.5 mm LCP Proximal Humerus Plates and screws from DePuy Synthes^®^. In 35 cases, a standard plate with 3 shaft holes was used, while in 10 cases, a long plate with 5 shaft holes was used.

In the study group, when a bone gap of at least 1 cm^3^ was identified after fracture reduction, an ENGIpore^®^ SH bone substitute was implanted. In 30 patients, the small SH format was used, whereas in 15 patients the large SH format was used. In 25 patients, intraoperative shaping of the device was necessary to adapt it to the morphology of the bone defect. In the control group, the metaphyseal bone gap was not filled with a bone graft or substitute. The average duration of the surgical procedure was 70 ± 16 min for the study group and 65 ± 23 for the control group. These differences were not statistically significant.

At the end of the procedure, a Gilchrist bandage maintained for the first week was placed. It was later replaced with an arm sling. The average hospitalization time was 2.5 days. At the end of hospitalization, the patients were discharged with the appropriate indications. Postoperative management consisted of keeping the operated limb protected until removal of the surgical sutures, approximately 15 days after the procedure. Thereafter, gentle elbow mobilization and Codman pendulum exercises were initiated. At approximately 30 days postoperatively, if clinical and radiographic evaluations revealed no complications, the sling was discontinued and the rehabilitation program for recovery of joint range of motion and muscle trophism was commenced [15].

Of the total study population, 41 patients completed the 12-month follow-up, while 4 patients underwent their final evaluation at approximately 3 months postoperatively. The mean follow-up duration was 10.25 ± 1.35 months. In the control group, 38 patients completed the 12-month follow-up, while 2 patients discontinued follow-up at 6 months, resulting in a mean follow-up of 11.7 ± 0.8 months.

**Figure 4:**
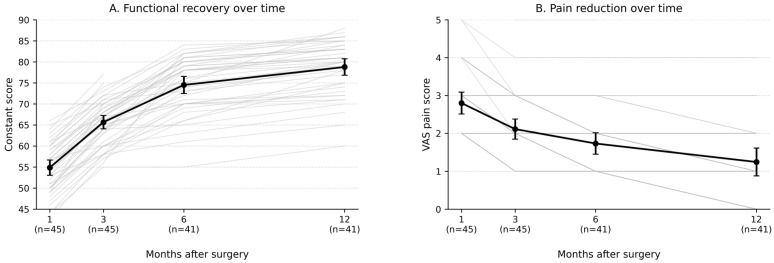
Longitudinal evolution of the primary clinical outcomes for the study group. Panel (**A**) shows the Constant score and panel (**B**) the VAS pain score at 1, 3, 6, and 12 months after surgery. Gray lines represent individual patient trajectories, whereas black circles and error bars indicate mean values with 95% confidence intervals. The sample size at each follow-up time point is reported below the *x*-axis.

**Figure 5:**
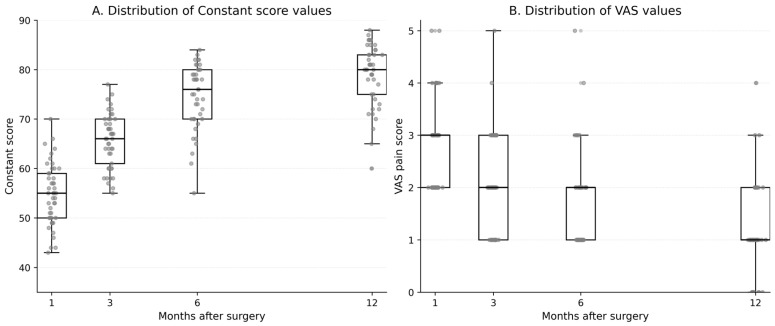
Distribution of observed values across follow-up for the study group. Boxplots show the distribution of the Constant score (panel (**A**)) and VAS pain score (panel (**B**)) at 1, 3, 6, and 12 months after surgery. Boxes indicate the interquartile range, the central line represents the median, whiskers indicate the range, and individual observations are overlaid.

During the follow-up, performed at intervals of 1, 3, 6 and 12 months, a functional evaluation was carried out using the Constant–Murley score (CMS) (Figure 4 and Figure 5). The CMS showed an approximately normal distribution at the assessed follow-up time points. Accordingly, CMS values are reported as mean ± SD. In the study group, CMS progressively improved from 54.87 ± 6.20 points at 1 month to 65.64 ± 5.43 points at 3 months, 74.51 ± 6.68 points at 6 months, and 78.78 ± 6.37 points at 12 months.

In the control group, CMS progressively improved from 55.77 ± 3.81 points at 1 month to 67.44 ± 6.01 points at 3 months, 73.44 ± 5.33 points at 6 months, and 79.23 ± 4.29 points at 12 months.

VAS pain scores showed a non-normal distribution and are therefore reported as median [IQR]. In the study group, VAS decreased from 3 [IQR: 2–3] at 1 month to 2 [IQR: 1–3] at 3 months, 2 [IQR: 1–2] at 6 months, and 1 [IQR: 1–2] at 12 months.

In the study group, VAS decreased from 2 [IQR: 1–3] at 1 month to 2 [IQR: 1–2] at 3 months, 2 [IQR: 1–3] at 6 months, and 1 [IQR: 1–2] at 12 months.

Longitudinal clinical outcomes are reported in Table 2. Both groups showed progressive functional improvement throughout follow-up. In the study group, the Constant–Murley Score (CMS) increased from 54.87 ± 6.20 at 1 month to 65.64 ± 5.43 at 3 months, 74.51 ± 6.68 at 6 months, and 78.78 ± 6.37 at 12 months. A similar improvement was observed in the control group, with CMS increasing from 55.77 ± 3.81 at 1 month to 67.44 ± 6.01 at 3 months, 73.44 ± 5.33 at 6 months, and 79.23 ± 4.29 at 12 months.

Linear mixed-effects modelling demonstrated a significant effect of time on CMS (*p* = 0.03), indicating progressive functional recovery during follow-up. Conversely, the main effect of group was not significant (*p* = 0.11), and no significant group-by-time interaction was observed (*p* = 0.34). These findings indicate that CMS improved significantly over time, while the magnitude and trajectory of functional recovery were comparable between the study and control groups. Post hoc within-group comparisons confirmed a significant CMS improvement in the study group from 74.51 ± 6.68 at 6 months to 78.78 ± 6.37 at 12 months (mean difference = +4.27 points, *p* = 0.02).

Pain also decreased progressively over time in both groups. In the study group, VAS decreased from 2.80 ± 0.99 at 1 month to 2.11 ± 0.91 at 3 months, 1.73 ± 0.92 at 6 months, and 1.24 ± 1.20 at 12 months. In the control group, VAS decreased from 1.98 ± 0.83 at 1 month to 1.87 ± 0.37 at 3 months, 1.65 ± 0.77 at 6 months, and 1.35 ± 0.55 at 12 months.

The mixed-effects model showed a significant effect of time on VAS (*p* < 0.05), indicating a significant reduction in pain during follow-up. The main effect of group was not significant (*p* = 0.10), and the group-by-time interaction was not significant (*p* = 0.70). Post hoc analyses confirmed a significant pain reduction in the study group from 1.73 ± 0.92 at 6 months to 1.24 ± 1.20 at 12 months (mean difference = −0.49 points, *p* < 0.05).

At single follow-up time points, CMS values did not differ significantly between the study and control groups at 1, 3, 6, or 12 months. VAS scores were significantly higher in the study group at 1 month compared with the control group (2.80 ± 0.99 vs. 1.98 ± 0.83, *p* = 0.001), whereas no significant differences were observed at later follow-up assessments. Sensitivity analysis restricted to patients with complete follow-up data confirmed a significant time-related change for both CMS and VAS (Friedman test: CMS, *p* = 0.02; VAS, *p* = 0.01).

Clinical and radiographic adverse events were recorded during follow-up and evaluated in relation to the presence of the biomaterial device. Data are reported for the final evaluable populations of 41 patients in the study group and 38 patients in the control group, respectively (Table 3). In the study group, there were three cases (7.3%) of poor consolidation, two cases (4.9%) radiographic signs of initial necrosis of the humeral head, and three cases (7.3%) of partial secondary fracture breakdown. In the control group, there were two cases (5.2%) of poor consolidation, two cases (5.2%) of radiographic signs of initial necrosis of the humeral head, and five cases (13.2%) of partial secondary fracture breakdown.

For delayed union, the *p*-value was 1.0, whereas for secondary breakdown the *p*-value was 0.47. No adverse event observed in the scaffold group was considered directly attributable to the implanted biomaterial.

In the study population, we observed three patients who required prosthetic replacement surgery (reverse shoulder arthroplasty) due to severe pain and poor functional capacity.

**Figure 6:**
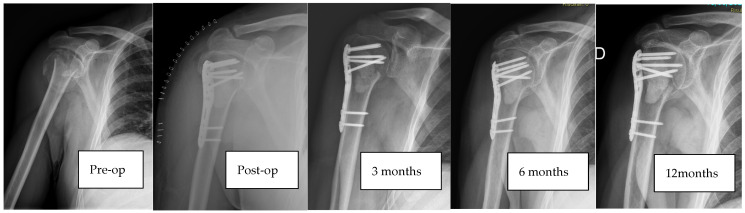
Representative radiographic follow-up of uncomplicated cases. Serial radiographs of representative patients from the study and control groups are shown preoperatively, immediately after surgery, and at 3, 6, and 12 months of follow-up. Radiographs demonstrate maintenance of reduction, stable implant positioning, progressive fracture consolidation, and absence of radiographic complications, including secondary displacement, screw penetration, implant failure, humeral head collapse, or device migration.

In the control group, two patients required conversion to reverse shoulder arthroplasty for the same clinical reasons. Although the scheduled clinical and radiographic follow-up was limited to 12 months, patients were subsequently recontacted to collect information on any further surgical procedures. This additional information revealed that three patients in the study group underwent conversion to reverse shoulder arthroplasty at 11, 13, and 15 months, respectively, while two patients in the control group underwent conversion at 15 and 18 months, respectively. Radiographic adverse events occurred in both groups, including delayed consolidation or early radiographic signs of humeral head osteonecrosis and partial secondary loss of reduction. No statistically significant differences were observed between groups for delayed consolidation/early osteonecrotic changes or secondary loss of reduction. Importantly, no cases of device migration, fragmentation, or unexpected resorption were observed in the scaffold group (Figure 6).

## 4. Discussion

There is increasing interest in the use of bone substitutes in trauma surgery. The availability of a ready-to-use device with reliable biomechanical properties makes these products particularly valuable in the operative setting [16]. In certain anatomical regions, such as the humeral head, the presence of a bone gap can compromise the biomechanics of osteosynthesis and negatively affect clinical outcomes [17].

Compared with autologous bone grafting, which remains the current gold standard, synthetic bone substitutes offer several practical advantages, particularly the avoidance of donor-site morbidity [18]. However, the cost-effectiveness of these products relative to autologous grafts remains debated [19,20].

Several techniques have been described for the management of proximal humerus fractures [21]. In our experience, the use of these devices demonstrated utility and versatility in the management of bone defects in the metaepiphyseal region of the humerus. In multiple cases, the fracture pattern involved coronal-plane collapse of the humeral head associated with fragmentation of the tuberosities and surgical neck. Following fracture reduction, a bone gap was consistently evident, necessitating the application of a biomimetic bone graft.

The utility and properties of biomimetic grafts were previously investigated by Knapp G. et al. [22] in a retrospective case–control study of 290 patients with acute fractures. A calcium phosphate bone graft was applied in 136 patients, whereas 154 patients served as controls. Fracture sites included the proximal humerus, distal radius, and proximal ulna. The authors concluded that these devices demonstrated both safety and efficacy, reducing complication rates during mid-term follow-up. These findings are consistent with our observations. In our case series, we observed a numerical reduction, compared with control group, in secondary breakdown, although this did not reach statistical significance.

The ready availability of the device did not prolong operative time and required no additional surgical field preparation. Our experience aligns with the review by Mondal S. et al. [23], which highlighted the intraoperative handling advantages of such substitutes. Indeed, the comparison between the two groups did not show statistically significant differences in terms of the duration of the surgical procedure.

The choice to use the SH configuration of the ENGIpore^®^ graft was determined by its geometry, which facilitates application in bone defects typical of the proximal humerus. The availability of different sizes (small, medium, and large) optimized product usage and reduced waste. Another relevant feature—confirmed in daily practice—is the importance of graft shape and structural design. As noted by Feng P. et al. [24], these factors are critical functional characteristics. The device used in our cohort was specifically conformed for the humeral head, improving adaptation at the fracture site and optimizing graft performance. This feature is essential because bone gaps may be small and require undersized grafts [25]. Nevertheless, in 25 procedures, intraoperative shaping of the device was still necessary to ensure optimal fit, regardless of the surgical approach used. This modification did not compromise the biological or mechanical properties of the device. The manufacturer guarantees preservation of biomechanical performance following shaping, and as described by Gillman C. E. et al. [26], the material demonstrates biological properties like natural bone with excellent biocompatibility. Its long persistence—reported to exceed three years—supports appropriate fracture remodeling and biomechanical stability. Although our follow-up extended to 12 months, no early degradation of the biomaterial was observed.

Clinical assessment during follow-up showed no statistically significant differences between the two groups about joint function and mid-term clinical symptoms. The only difference was observed in short-term pain symptoms, as measured by the VAS, with the study group reporting worse outcomes, namely greater pain. In our opinion, this finding may be explained by the likely need to perform more complex reduction maneuvers to manage the fracture and fill the bone gap in the group in which the device was implanted. Moreover, this difference was no longer statistically significant as follow-up progressed.

Current literature supports the safety profile of these biomaterials [27,28]. During follow-up, particular attention was paid to monitoring the graft and any potential adverse events. We evaluated signs of infection, biomechanical complications, device shrinkage, and dislocation. These parameters served as indicators of potential synthesis instability, which could compromise healing and lead to humeral head collapse. No cases of device mobilization or resorption were identified during the study period. Radiographic and clinical adverse events were observed in both groups. In the scaffold group, three patients showed poor consolidation, two showed early radiographic signs of humeral head osteonecrosis, and three showed partial secondary loss of reduction. In the control group, two patients showed poor consolidation, two showed early radiographic signs of humeral head osteonecrosis, and five showed partial secondary loss of reduction. No statistically significant differences were observed between groups for these outcomes. Importantly, no cases of device migration, fragmentation, or unexpected resorption were observed in the scaffold group, and no adverse event was considered directly attributable to the implanted biomaterial.

The study population consisted of patients with complex proximal humerus fractures associated with metaepiphyseal bone defects. This condition complicates biomechanical stabilization and fixation, making it suitable for assessing device performance. Although identifying optimal endpoints in this context is challenging, evaluation of osteosynthesis stability and fracture healing provides indirect evidence supporting the validity of these biomaterials [29].

The present study demonstrates that patients treated with plate osteosynthesis experienced a significant and progressive clinical improvement over the 12 months following surgery, accompanied by a reduction in pain intensity. Functional recovery, as assessed by the Constant score, was substantial during the early postoperative period, particularly within the first 3 to 6 months, but remained significant between 6 and 12 months, suggesting that recovery continues beyond the initial rehabilitation phase. Likewise, VAS scores showed a steady decline throughout the follow-up period. These findings are consistent with a favorable postoperative recovery pattern after locking-plate fixation, with progressive functional improvement and pain reduction in both groups. However, no significant between-group differences in CMS were observed, and the group-by-time interaction was not significant, suggesting comparable recovery trajectories between groups. Nevertheless, the results should be interpreted in light of several limitations, including the observational design, incomplete follow-up at later time points, and the subjective nature of pain assessment.

The number of surgical revisions involving reverse shoulder arthroplasty was small, with three cases in the scaffold group and two cases in the control group. The limited sample size and the multiple variables associated with proximal humerus fractures do not allow this finding to be considered statistically meaningful. Kristensen et al. [30] reported a 1.5-fold higher risk of prosthetic replacement in patients treated with osteosynthesis, concluding that the risk of requiring arthroplasty following internal fixation failure should be carefully considered in therapeutic decision-making.

It is important to emphasize, however, that the use of biomaterials cannot substitute the fundamental biomechanical principles of proper osteosynthesis, which remain essential for achieving successful outcomes. This retrospective cohort study suggests that adjunctive use of a porous hydroxyapatite scaffold during locking-plate fixation of proximal humerus fractures with metaphyseal bone void is feasible and was not associated with obvious device-related complications during follow-up. However, because of the retrospective non-randomized design, potential selection bias, limited sample size, and incomplete follow-up, the study does not allow for definitive causal inference regarding the independent efficacy of the biomaterial. In our cohort, we observed encouraging clinical and radiographic findings, consistent with the available literature; however, these data should be interpreted as supportive of feasibility and short- to mid-term safety rather than proof of device efficacy [31,32].

### Limitations and Advantages of the Study

The study was conducted on a small number of patients, which limits the strength of the conclusions that can be drawn. Additional limitations include the retrospective nature of the study and the medium-term follow-up that may not capture complications with late onset. A potential selection bias should be acknowledged, as the decision to use scaffold augmentation was based on intraoperative assessment of the metaphyseal bone defect and surgeon judgment rather than random allocation. The strengths of the study include the use of a standardized fixation system, the same surgical team, and a consistent postoperative rehabilitation protocol. To achieve more significant results, it would be necessary to increase the number of patients included in the study and further extend the follow-up period.

## 5. Conclusions

In this retrospective comparative cohort study, the adjunctive use of a porous hydroxyapatite bone substitute during locking-plate fixation of proximal humerus fractures with metaphyseal bone loss was feasible and was not associated with evident device-related complications during short- to mid-term follow-up. Both the scaffold and control groups showed progressive functional recovery and pain reduction over time, with comparable clinical trajectories and no significant between-group differences in CMS at any follow-up time point. These findings support the apparent safety of scaffold augmentation in selected cases, but do not demonstrate clear clinical superiority over locking-plate fixation alone. Owing to the retrospective non-randomized design, limited sample size, potential selection bias, and incomplete follow-up, further prospective controlled studies with longer follow-up are needed to clarify the independent efficacy of the biomaterial.

## 6. Future Directions

The increasing use of biomimetic bone graft substitutes may allow for the development of larger patient cohorts and more robust comparative analyses. Future studies should include longer follow-up to better assess late complications, graft remodeling, and the long-term clinical relevance of scaffold augmentation in proximal humerus fractures.

## Figures and Tables

**Table 1:** Summary of main data from the population under investigation.

Variable	Study Group with Device (45)	Control Group (40)	*p*-Value
Age, years	67.0 ± 8.7	65.0 ± 7.5	0.41
Female/male sex	27/18	23/17	0.61
ASA	2.84	2.77	0.82
Right/left side	23/22	21/19	0.51
Dominant side	25	21	0.43
AO/OTA class	(25) 11B; (20) 11C	(22) 11B; (18) 11C	0.91
Neer class	(24) 3-part; (21) 4-part	(21) 3-part; (19) 4-part	0.87
Surgical approach	(27) DP *; (18) TD *	(22) DP *; (18) TD *	0.77
Surgical procedure time	70 ± 16 min	65 ± 23 min	0.13

* DP= deltoid–pectoral approach; TD = trans-deltoid approach.

**Table 2:** Clinical outcomes at different follow-up time points in the study and control groups. CMS values are reported as mean ± standard deviation because they showed an approximately normal distribution according to the Shapiro–Wilk test; between-group comparisons were performed using Student’s *t*-test for independent samples. VAS values are reported as median [interquartile range] because they showed a non-normal distribution; between-group comparisons were performed using the Mann–Whitney U test. Longitudinal changes were analyzed using mixed-effects models, with non-parametric sensitivity analysis for complete cases.

FU (Months)	No. of Patients Study Grp; Control Grp	CMS Study Grp; Control Grp (*p*-Value)	VAS Study Grp; Control Grp (*p*-Value)
1	45; 40	54.87 ± 6.20; 55.77 ± 3.81 (*p* = 0.42)	3 [2–3]; 2 [1–3] (*p* = 0.001)
3	45; 40	65.64 ± 5.43; 67.44 ± 6.01 (*p* = 0.15)	2 [1–3]; 2 [1–2] (*p* = 0.11)
6	41; 40	74.51 ± 6.68; 73.44 ± 5.33 (*p* = 0.43)	2 [1–2]; 2 [1–3] (*p* = 0.67)
12	41; 38	78.78 ± 6.37; 79.23 ± 4.29 (*p* = 0.71)	1 [1–2]; 1 [1–2] (*p* = 0.60)

**Table 3:** Radiographic and clinical adverse events during follow-up. Data are reported as n/N (%). *p*-values were calculated using Fisher’s exact test. Conversions to reverse shoulder arthroplasty were collected through additional patient recontact after the scheduled 12-month follow-up. The denominator refers to patients for whom extended follow-up information was available.

Radiographic/Clinical Event	Study Group, n/N (%)	Control Group, n/N (%)	*p*
**Poor consolidation**	3/41 (7.3%)	2/38 (5.2%)	1.00
**Early radiographic signs of humeral head osteonecrosis**	2/41 (4.9%)	2/38 (5.2%)	1.00
**Partial secondary fracture breakdown**	3/41 (7.3%)	5/38 (13.2%)	0.47
**Device migration**	0/41 (0%)	—	—
**Device fragmentation/resorption**	0/41 (0%)	—	—
**Screw cut-out/articular penetration**	0/41 (0%)	0/38 (0%)	Not applicable
**Infection**	0/41 (0%)	0/38 (0%)	Not applicable
**Conversion to reverse shoulder arthroplasty**	3/41 (7.3%)	2/38 (5.2%)	1.00

## Data Availability

The data presented in this study are available on request from the corresponding author.
